# Rheumatoid Arthritis-Like Symptoms After Taking Relugolix, With Primary Exacerbation After Discontinuation of the Drug

**DOI:** 10.7759/cureus.53584

**Published:** 2024-02-04

**Authors:** Shohei Tanabe, Kazuyuki Tsuboi

**Affiliations:** 1 Obstetrics and Gynecology, Kobe City Medical Center West Hospital, Kobe, JPN; 2 Collagen Disease and Rheumatology, Kobe City Medical Center West Hospital, Kobe, JPN

**Keywords:** pain management, joint symptom, estrogen level, • rheumatoid arthritis, relugolix

## Abstract

We report a case of a 43-year-old woman who developed rheumatoid arthritis-like symptoms after taking relugolix, presenting a diagnostic challenge in distinguishing between initial symptoms of rheumatoid arthritis and the side effects of the drug. The patient, scheduled for a total laparoscopic hysterectomy owing to uterine fibroids, started taking relugolix five and a half months prior to surgery. Three months later, she developed rheumatoid arthritis-like stiffness in both hands, especially in the mornings. Despite consultations with the rheumatology department and negative blood and imaging findings for rheumatoid arthritis, her joint symptoms worsened after surgery. Treatment for early-stage rheumatoid arthritis was initiated, and the symptoms peaked after six months. Similar to estrogen-lowering aromatase inhibitors that are known to cause joint symptoms, relugolix might also induce these effects.

## Introduction

Relugolix, a gonadotropin-releasing hormone antagonist, was approved by the US Food and Drug Administration in May 2021 as an oral medication for treating heavy menstrual bleeding due to uterine fibroids [1]. Known to significantly reduce menstrual blood while preserving bone density, relugolix is also used preoperatively to reduce the uterine volume [2]. Its side effects include hypoestrogenic symptoms, such as hot flashes, insomnia, and headaches [3]. Rheumatoid arthritis, characterized by joint pain and stiffness, commonly affects women over the age of 40 [4], and early treatment in consultation with a specialist is crucial to prevent joint destruction and deformation [5]. We report a case of rheumatoid arthritis-like symptoms developing after pre-surgery use of relugolix and worsening after surgery, posing diagnostic challenges.

## Case presentation

**Figure 1:**
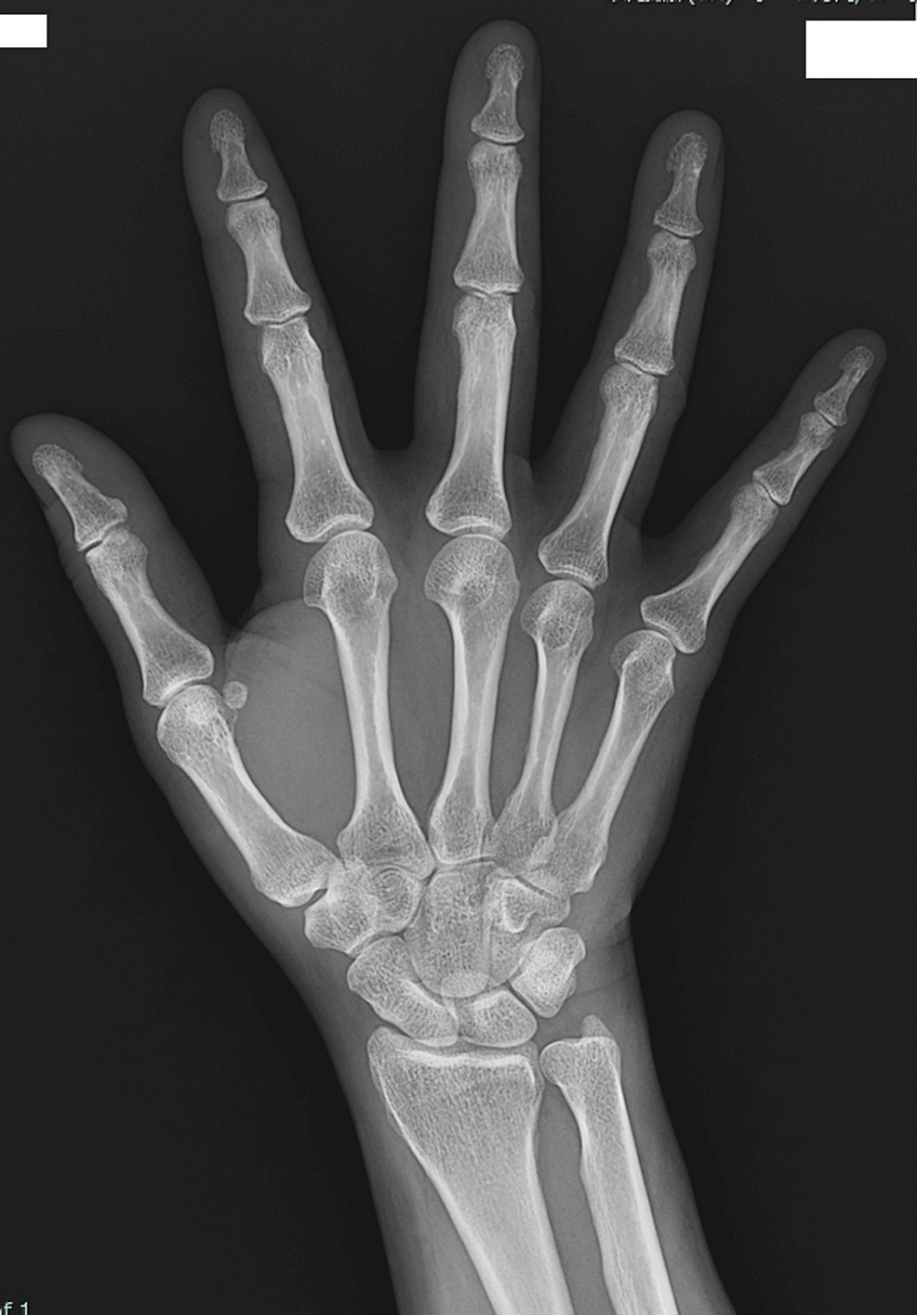
X-ray of the right hand No findings suggestive of a rheumatoid joint were noted.

**Figure 2:**
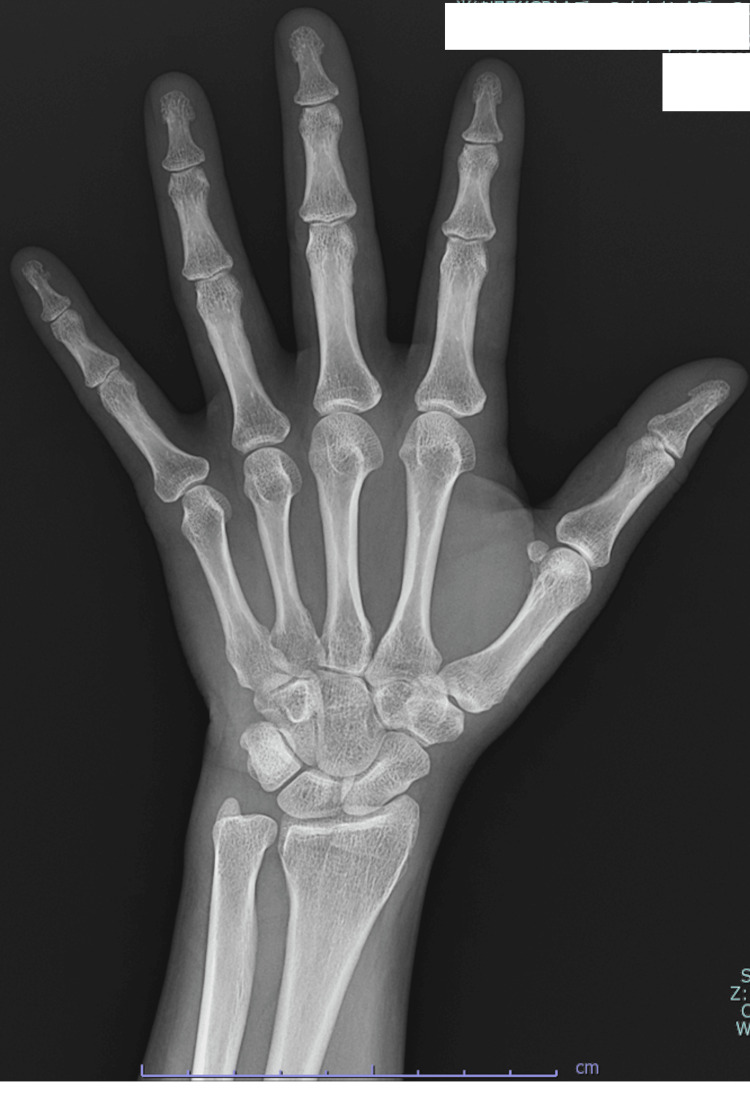
X-ray of the left hand No findings suggestive of a rheumatoid joint were noted.

A 43-year-old woman was referred for uterine fibroid surgery. She had a history of one pregnancy, delivered by cesarean section, and previous consultations for right hip pain. Magnetic resonance imaging revealed multiple fibroids. She started taking relugolix 169 days before surgery. After approximately three months, she experienced increased morning stiffness in both hands, prompting consultation with our rheumatology department. Although her family history was negative for rheumatoid arthritis, her mother was suspected of having some collagen disease. Despite the possibility of rheumatoid arthritis, hand joint X-rays (Figures 1, 2) and ultrasound showed normal findings. Various autoantibodies were tested, with only antinuclear antibody being slightly above normal, but no conclusive evidence of any collagen disease was found (Table 1). Relugolix was discontinued the day before surgery. The patient underwent a total laparoscopic hysterectomy, with a surgery time of 150 minutes and no blood loss. She was discharged on postoperative day 3. At one-month follow-up, she reported increased hand joint stiffness and daily non-steroidal anti-inflammatory drug use. Rheumatoid arthritis was considered, and tests for erythrocyte sedimentation rate and matrix metalloproteinase-3 were conducted monthly for three months. All test results were negative (Table 2), as was that for C-reactive protein outside the perioperative period. Treatment with iguratimod 25 mg/day was initiated and later increased to 50 mg/day without symptom improvement. Upper body joint pain and insomnia ensued, for which additional treatment with methotrexate 6 mg/week was initiated for six months post-symptom onset. Subsequently, her pain improved and her insomnia was resolved.

**Table 1:** Test results of autoantibodies performed at our Department of Rheumatology and Collagen Diseases Test results of autoantibodies performed at our Department of Rheumatology and Collagen Diseases. The results were not suspicious for rheumatoid arthritis or collagen disease.

Laboratory values		Reference range
IgG	1217	870-1700
C3	92	65-135
C4	15	13-35
Antinuclear antibody	40	<40
Homogeneous	(-)	-
Speckled	(+)	-
Nucleolar	(-)	-
Peripheral	(-)	-
Centromere	(-)	-
Discrete N dots	(-)	-
Nuclear envelope	(+)	-
Double-stranded DNA IgG	1.6	<12.0
Anti-centromere antibody	<1.0	<10.0
Anti-ribonucleoprotein antibody	<2.0	<10.1
Anti-SS-A antibody	<1.0	<10.2
Rheumatoid factor	<3	<15
Anti-ccp antibody	<0.5	<4.5
Lupus anticoagulant	0.97	<1.3
Anti-Clβ2GP1 antibody	<0.7	<3.5
Anti-cardiolipin antibody	<4.0	<12.3

**Table 2:** MMP-3 antibodies and erythrocyte sedimentation rate measured monthly for 3 months after surgery The blood sample results remained normal.

	MMP3 (ng/ml)	Erythrocyte sedimentation rate (mm/h)
Postoperative 1 month	40.6	7.0
Postoperative 2 months	40.7	3.0
Postoperative 3 months	44.3	3.0
Reference range	17.3-59.7	3.0-15.0

## Discussion

In this case, the patient suffered from symptoms resembling rheumatoid arthritis, and distinguishing whether these were due to an existing condition or a side effect of relugolix was challenging. Early referral and treatment initiation are recommended for suspected rheumatoid arthritis [6]. Even though this patient did not meet the 2010 EULAR criteria for rheumatoid arthritis diagnosis [7], treatment was initiated considering the risks of delayed treatment. Whether the improvement was due to rheumatoid arthritis treatment or the cessation of relugolix remains unclear.

Joint symptoms as a side effect of relugolix are not commonly known. However, joint symptoms are a known side effect of aromatase inhibitors, being attributed to estrogen reduction [8]. This condition is known as Aromatase Inhibitor-Associated Musculoskeletal Syndrome (AI-AMS) [9]. Joint symptoms associated with AI-AMS typically manifest in various parts of the body, including the hands, feet, and hips, and are often accompanied by early morning stiffness and difficulty sleeping [10].

The symptoms observed in the current case are strikingly similar; relugolix, known to induce a hypoestrogenic state, might therefore lead to joint symptoms akin to those caused by aromatase inhibitors. In this case, symptoms peaked at six months. This timeline aligns with reports indicating that symptoms of aromatase inhibitor-induced joint pain can peak around the six-month mark [11].

Treatment options for AI-AMS typically involve pharmacotherapy, including vitamin D and omega-3 fatty acids, as well as exercise therapy. However, systematic reviews have struggled to conclusively evaluate these treatments due to the heterogeneity in research methodologies [12]. Similarly, systematic reviews of exercise therapy have not demonstrated definitive efficacy [13].

Consequently, in cases like the current one, where joint symptoms emerge due to relugolix, presenting an effective treatment remains challenging. Continued research and further reports on effective treatments are eagerly anticipated.

## Conclusions

We have experienced a case in which rheumatoid arthritis-like joint symptoms may have appeared as a side effect of relugolix. The patient's joint symptoms were more likely to have been a side effect of relugolix, as there were no laboratory results to support rheumatoid arthritis. The joint symptoms improved after six months.

In the future, for patients who develop rheumatoid arthritis-like joint symptoms after relugolix treatment, it is important to consult a specialist at an early stage to discuss treatment, as demonstrated in this case.
